# Effects of behavioural change communication (BCC) on menstrual hygiene practices among urban school adolescent girls: a pilot study

**DOI:** 10.1136/bmjnph-2023-000754

**Published:** 2023-11-14

**Authors:** Farzana Saleh, Kazi Rumana Ahmed, Taslima Khatun, Nandini Roy, Sadia Uddin, Md Rowshan Kabir

**Affiliations:** 1 Community Nutrition, Bangladesh University of Health Sciences, Dhaka, Bangladesh; 2 School of Health & Rehabilitation Sciences, The University of Queensland, Saint Lucia, Queensland, Australia; 3 Health Promotion & Health Education, Bangladesh University of Health Sciences, Dhaka, Bangladesh; 4 Public Health, National Cheng Kung University (NCKU), Tainan, Taiwan; 5 Nutrition, Dikoda Ltd, London, UK

**Keywords:** Nutritional treatment, Nutrient deficiencies

## Abstract

**Background:**

Research on menstrual hygiene management practices (MHMP) has yet to be conducted among adolescent girls in Bangladesh who have gained services from the Urban Primary Health Care Project (UPHCP). This study aimed to assess the effects of behavioural change communication activities on MHMP among urban school adolescent girls.

**Methods:**

A convenience sample of 270 adolescent girls (aged 10–19) who had no chronic diseases from 5 schools in Dhaka city was selected using a descriptive cross-sectional design from February to May 2018. A semistructured (interviewer-administered) questionnaire was used. Frequencies were calculated for descriptive analysis.

**Results:**

About 17% of girls had irregular menstrual cycles, 57% felt uneasy and 27% had >7 days of menstrual flow. Fifty-five per cent of the girls used sanitary napkins. Most (95%) and 26% of the girls did not change their pads during school and at night, respectively. Sixty-five per cent of girls disposed of the used pads at the public dustbin, and 83% bathed during menstrual. Only 4% of girls were aware of the iron folic acid tablets.

**Conclusions:**

Despite the availability of services from UPHCP, the acceptance and adherence to MHMP among adolescent girls still need to be improved.

## Introduction

Adolescence is a time of transition involving multidimensional changes characterised by first menstruation, a natural and beneficial biological event, and significant physical, emotional, cognitive and social changes.^1 2^ Most societies consider menstruation to be taboo.^1–3^ Improper knowledge, lack of preparation and poor practices are the key obstacles to appropriate management during menstruation.^4^

Mothers and other female relatives are our country’s primary sources of information on menstruation.^5 6^ Understanding adolescent girls’ menstrual hygiene management practices (MHMP) is crucial to promote sustainable goals 3, 4, 5 and 6.^7^ Different studies^1–3 8–12^ found unhealthy menstrual management practices among Bangladeshi adolescent girls and recommended health education programmes. Despite being a significant public health issue, more understanding is needed of the proper management of menstrual hygiene in low-income and middle-income countries such as Bangladesh.^13^ The rapid expansion has placed significant pressure on health services and facilities in urban areas. Bangladesh has a robust public sector primary healthcare network system in the rural areas, though there needs to be more similar arrangements in the urban areas. One of the goals of the Urban Primary Health Care Project (UPHCP) is to improve adolescents’ health. UPHCP has an adolescent corner for adolescent counselling, and under UPHCP activities, adolescent girls have received behavioural change communication (BCC) messages on teenage pregnancy, anaemia and hygiene practices. Therefore, this study assessed the effects of BCC activities on MHMP among urban school adolescent girls with access to primary healthcare services.

## Methodology

This descriptive cross-sectional study used a convenience sampling technique and took place between February and May 2018 in the Mohammadpur area of Dhaka city. We obtained permission from the authorities and selected a total of 270 adolescent girls, aged 10–19, from 5 schools.

Girls who had any chronic diseases were excluded. The minimum required sample size was calculated using the formula n=z^2^pq/d^2^ (where z=1.96, p=the proportion of severe undernourished urban adolescent girls was 22.4%,^14^ q=1 p and d=allowable error of known prevalence, ie, 5%), considering non-response of 5% of the total number of adolescent girls. A two-part semistructured (interviewer-administered) questionnaire was used in the study. Part 1 described the adolescent girls’ sociodemographic data, including age, religion, education, occupation, education level of the parents and monthly income of the family. Part 2 had information about menstruation and hygiene practices during menstruation. A pretest was run before the questionnaire was finalised. Frequencies were calculated for descriptive analysis. The SPSS software for Windows V.26 (SPSS) was used for analysis. The classification of socioeconomic status was made according to the per capita Gross National Income (GNI) 2006 and according to World Bank calculations.^15^

## Results and discussion

The mean age of the adolescent girls in this study was 13.9±1.5 years. Most (90%) of the girls lived with their parents. One-third of girls’ parents were illiterate or primarily passed. More than half (57%) of girls’ fathers were workers, and 74% of mothers were homemakers. Most (66%) of the girls were from lower-middle-class families, and 57% had no attached bathroom.

The mean menarche age of the girls was 11.8±0.91 years. Of them, 70% knew about menstruation from their mother, which was higher than other studies.^11 12^ Out of our respondents, only 10% reported learning from school teachers, which is lower than the findings in Ha and Alam^11^ and higher than those in Alam *et al’s* study.^6^

In this study, 17% of adolescents had irregular menstrual cycles, similar to other studies,^9 12^ 57% felt uneasy during the cycle, 27% had more than 7 days duration of menstrual flow, and these were comparable with Parvin *et al*.^9^ However, an intervention study^8^ explored that after receiving a menstrual educational programme, there was a significant improvement among adolescent girls in the regularity of the menstrual cycle, less pain and not as much excessive bleeding. In contrast, urban adolescent girls in our study faced these problems frequently after primary healthcare counselling (table 1).

**Table 1:** Pattern of the menstrual cycle and hygienic practices among adolescent girls (n=270)

Variables	n (%)
Pattern of menstrual cycles	
Menstrual cycle	
Irregular	46 (17)
Feeling uneasy during the cycle	
Pain	154 (57)
Duration of menstrual flow (days)	
≥7	72 (27)
Menstrual hygiene management practices	
Types of material used during the menstrual cycle	
Non-disposable linen	121 (45)
Sanitary napkins	149 (55)
Reuse the pads	
Yes	66 (24)
Changes pads during school	
No	257 (95)
Changes pads during the night	
No	70 (26)
Disposal of used materials	
Indiscriminate through	74 (27)
Throw in the public dustbin	174 (65)
Flush it	22 (8)
Taking a bath during the time	
Yes	223 (83)
Cleaning of external genital	
No	4 (2)

**Figure 1:**
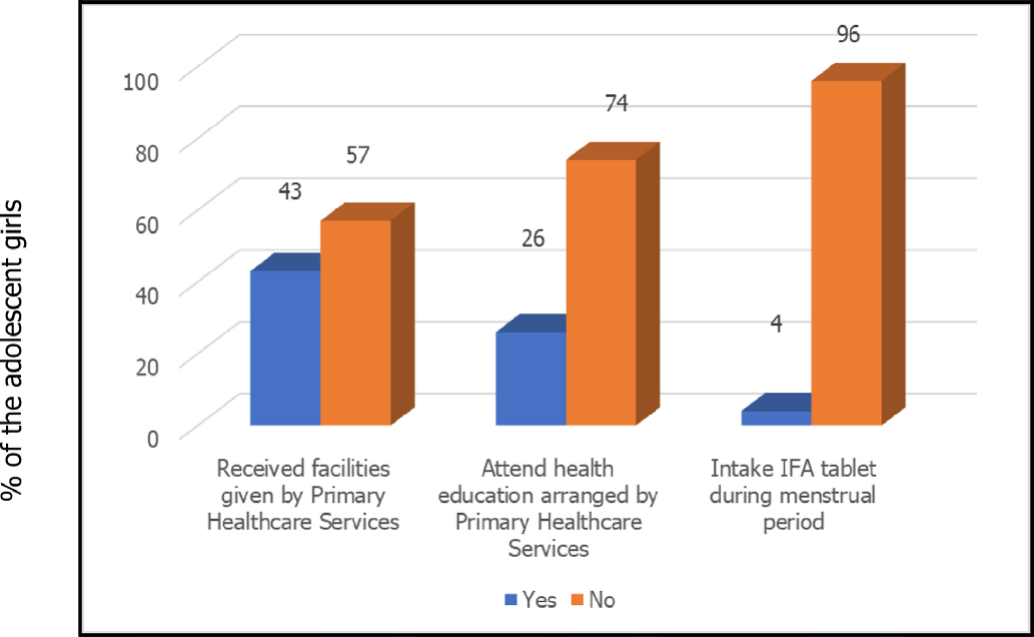
Distribution of adolescent girls according to the health facilities and health education given by primary healthcare services (n=270).

In this study, we examined whether adolescent girls participated in the counselling programme offered by UPHCP and received knowledge about iron-folic acid (IFA) tablets and health facilities from the centre. The findings were quite surprising. Most girls (74%) did not attend the counselling programme arranged by UPHCP, and 57% did not receive any facilities (figure 1). Despite these challenges, 55% of the girls in our study used sanitary napkins, consistent with other studies’ findings.^8 12^ but higher than the results reported in studies by Parvin *et al*,^9^ Ha and Alam^11^ and Alam *et al*,^6^ Moreover, approximately 76% used pads only once, and 83% practised bathing during their menstrual period (table 1). These commendable practices can be attributed to the fact that 70% of our study participants received information from their mothers, while 10% received information from their school teachers.

However, there were still areas of concern regarding menstrual hygiene management among adolescent girls. Around 45% of the girls had not yet used sanitary napkins. The majority of adolescents (95%) did not change their pads during school hours, 26% did not change pads at night and 65% of the girls disposed of used pads by throwing them into public dustbins, which is unhygienic. Additionally, 24% continued to reuse the pads (table 1). In this study, only 2% of the girls did not clean their external genital organs, which is acceptable. These findings are consistent with results from various other studies.^6 9 11 12^

A notable finding is that 96% of individuals had no knowledge of IFA tablets (figure 1). About 87% of girls did not know about the benefits of IFA tablets, and 8% of mothers or caregivers did not allow girls to take IFA during menstruation. Unmarried adolescent girls in our country do not have access to healthcare. Bangladesh Government has created UPHCP for this purpose. However, from the above discussion, it is identified that despite providing health education from the primary healthcare services, practices were not acceptable among urban adolescents. They need to be made aware of healthy practices. Probable reasons are that parents of the adolescents did not understand the importance of primary healthcare services. The study had some limitations. This was a preliminary study utilising a cross-sectional design, and it did not allow for establishing causal inferences. The sample size was relatively modest, and data collection was conducted via interviewer-administered questionnaires, which may introduce subjective variations.

A robust monitoring system might have become one of the solutions for improving the situation. Repeated strengthening of health education and strong motivation will bring about positive changes in hygiene practices regarding menstrual management. While the current study might not provide a comprehensive overview of the nation’s adolescent MHMP landscape, it does, however, illuminate the circumstances faced by the underprivileged demographic.
